# Preparation of Cemented Oil Shale Residue–Steel Slag–Ground Granulated Blast Furnace Slag Backfill and Its Environmental Impact

**DOI:** 10.3390/ma14082052

**Published:** 2021-04-19

**Authors:** Xilin Li, Kexin Li, Qi Sun, Ling Liu, Jianlin Yang, Haowen Xue

**Affiliations:** 1 School of Civil Engineering, Liaoning Technical University, Fuxin 123000, China; likexin202104@163.com (K.L.); sunqi@lntu.edu.cn (Q.S.); liuling@lntu.edu.cn (L.L.); xuehaowen226@163.com (H.X.); 2 College of Materials Science and Engineering, Liaoning Technical University, Fuxin 123000, China; jlyang@alum.imr.ac.cn

**Keywords:** cemented oil shale residue–steel slag–ground granulated blast furnace slag backfill, response surface method, microstructure, leaching test, environmental pollution

## Abstract

A new environmentally friendly cemented oil shale residue–steel slag–ground granulated blast furnace slag backfill (COSGB) was prepared using oil shale residue (OSR), steel slag (SS) and ground granulated blast furnace slag (GGBS) as constituent materials. Based on univariate analysis and the Box–Behnken design (BBD) response surface method, the three responses of the 28 days unconfined compressive strength (UCS), slump and cost were used to optimize the mix ratio. Using a combination of scanning electron microscopy-energy dispersive spectroscopy (SEM-EDS), Fourier transform infrared (FTIR) spectroscopy, X-ray diffraction (XRD) and mercury intrusion porosimetry (MIP), the reaction products, microscopic morphology and pore structure of the specimens with the optimal mix ratio at different curing ages were analyzed. The influence of heavy metal ions from the raw materials and the COSGB mixtures on the groundwater environment was studied by leaching tests. The research demonstrates that the optimal mix ratio is GGBS mixing amount 4.85%, mass ratio of SS to OSR 0.82, and solid mass concentration 67.69%. At shorter curing age, the hydration products are mainly calcium alumino silicate hydrate (C-A-S-H) and calcium silicate hydrate (C-S-H) gels. With the increase of curing age, ettringite (AFt) and C-S-H gels become the main source of the UCS. Meanwhile, the porosity of the filler decreases continuously. The leaching concentration of heavy metal ions from the COSGB mixtures is all lower than the leaching concentration of raw materials and meet the requirements of the Chinese groundwater quality standard (GB/T 14848-2017). Therefore, this new COSGB cannot pollute the groundwater environment and meets backfill requirements. The proposed technology is a reliable and environmentally friendly alternative for recycling OSR and SS while simultaneously supporting cemented paste backfill (CPB).

## 1. Introduction

A high concentration of cemented paste backfill (CPB) material, a mixture of tailings, coal gangue, ground granulated blast furnace slag (GGBS), construction waste and other solid wastes with cementitious materials, is used to fill goafs. CPB can control mining subsidence and utilize solid waste, so this material has become an important means of green mining [1]. On the one hand, ordinary Portland cement (OPC) is often used as a cementitious material in CPB materials, but the cost is high. Its cost generally accounts for 60% to 80% of the filling cost [2]. In China, the price of P.O42.5 OPC is approximately 0.0748 USD/kg, and the price of S95 GGBS is approximately 0.0141 USD/kg. Oil shale residue (OSR) and steel slag (SS) are common solid wastes with lower cost. It is an inevitable choice to use mine solid waste as a substitute for cement to reduce the cost of filling materials. On the other hand, manufacturing OPC requires the calcination of limestone at very high temperatures and accounts for 8% of global CO_2_ emissions [3]. Partially or completely replacing OPC with solid wastes can reduce carbon emissions in the climate emergency and the cost of CPB at the same time [4]. OSR and SS, as typical solid wastes, have not yet been effectively utilized. For example, the OSR in Fushun, Liaoning Province, China and the SS in Anshan, Liaoning Province, China are piled up like mountains. These solid wastes not only occupy land but also cause serious damage to the ecological environment. The OSR and SS stacked in open air under the action of rainwater leaching and water leaching can pollute the soil-groundwater system [2,5]. If OSR and SS are used as components in CPB, and GGBS less than 10% of the total solids is mixed in, a new CPB material can be prepared. Exploring the impact of this new CPB material on mine water environments is of great significance. In the meantime, it will provide a pathway to achieving a circular economy in mines [6].

Many scholars have carried out research and development on new CPB materials and the mechanical properties of CPB materials. Mangane et al. [7] used 20% cement and 80% GGBS as cementitious materials and mixed different types of water-reducing agents to prepare CPB. The results validated that the influence of superplasticizers on CPB performances depended on the type and dosage of the admixture. Polycarboxylate presented the best performances. Chen et al. [8] investigated the feasibility of recycling two different solid wastes, phosphogypsum (PG)and construction and demolition waste (CDW), as CPB materials. The results indicated that PG and CDW-based CPB could support an underground stope after failure. The proposed technology was a reliable and environmentally friendly alternative for recycling PG and CDW. Cihangir et al. [9] used alkali-activated neutral and acidic blast furnace slags (AASs) with liquid sodium silicate (LSS) and sodium hydroxide (SH) instead of cement to prepare CPB materials. The effects of sulfate and acid on the short- and long-term mechanical performance of CPB specimens were investigated. The authors claimed that the CPB specimens of alkali activated neutral slag (NS) gained early strength consistently at a slower rate than that of acidic slag irrespective of the activator type. SH-activated slag specimens developed higher 28-day strengths than LSS-activated slag specimens. Zheng et al. [10] reported that the complex incorporation of limestone powder (LP) and water-reducing admixture (WRA) remarkably improved the workability of CPB mixtures and increased the unconfined compressive strength (UCS) and the long-term stability of CPB specimens by reducing W/B ratio without decreasing the slump value. Li et al. [11] studied the effect of sulfate on the early age strength of the backfill with quartz tailings as aggregate, 50% cement and 50% blast furnace GGBS as cementitious materials. It was found that a comparatively high sulfate concentration led to strength reduction at all early ages. Sun et al. [12] prepared geopolymer cemented coal gangue-fly ash backfill by using alkali-activated fly ash as a cementitious material. This research was beneficial to waste utilization and cleaner production. Koohestani et al. [13] indicated that the addition of vinyl silane to CPB provided a higher UCS value and reduced the required amount of water for a specific slump height. However, the addition of vinyl and methyl organosilanes reduced the early strength development.

To analyse the micro hydration mechanism of CPB, Yan et al. [14] performed a study on the trend of the coupling effect of sulfate and temperature on the early hydration reaction and mechanical properties of CPB. It was found that both sulfate and temperature significantly affected the hydration process and thus influenced the internal volume change and mechanical properties of CPB. Yılmaz et al. [15] used a combination of mercury intrusion porosimetry (MIP) and X-ray diffraction (XRD) methods to study finely ground CDW as partial replacements for sulfide tailings on the microstructural properties of CPB. They reported that the use of CDW as partial replacements for sulfide tailings enhanced the strength properties of CPB specimens by decreasing the total and macro porosity. Sun et al. [16] used computed tomography (CT) and a small loading device to perform real-time uniaxial compression scan tests to obtain the two-dimensional CT images under different pressure conditions. The results showed that during the process of bearing, the shear deformation of microscopic void cell within CPB occurred under forces, eventually leading to the destruction of CPB. Liu et al. [17] used nuclear magnetic resonance (NMR) and scanning electron microscopy (SEM) to characterize the pore structure of CPB specimens. It could be observed that there was a negative exponential relationship between the UCS and porosity of CPB specimens and a linear inverse relationship between the UCS and fractal dimension. Hu et al. [18] used NMR and SEM to obtain the microscopic characteristics of a backfill specimen and established the relationship between the pore diameter of the backfill specimen and the UCS. They reported that the pore size of the CPB was mainly small, and the pore diameter of small pores was linearly and inversely proportional to the UCS of the specimens.

In terms of the environmental effect of CPB, Hu et al. [19] studied the toxicity of heavy metals in SS and its asphalt mixtures by physicochemical characterization, batch leaching tests and semi-dynamic tests to determine the migration capacity and leaching characteristics of heavy metals. The results presented that steel slag had a low pollution risk in short-term leaching, whereas the cumulative release mass of Cd, Ni, As and Pb had a certain environmental impact in the long-term leaching process. The stripping off of asphalt could aggravate the release potential of heavy metals from asphalt mixture, but the pollution risk remained controllable. Wang et al. [20] prepared a non-autoclaved aerated concrete using oil-based drilling cutting pyrolysis residues (ODPR) and fly ash instead of cement. The results showed that ODPR had a certain pozzolanic activity which could play the role of active materials. When ODPR served as recycled aggregates and admixture, it would not be the substance of environmental contamination. Li et al. [21] investigated the feasibility of using fly ash slag-based binders for mine backfill and its associated leaching risk. They found that the risk of hazardous constituent leaching was mainly controlled by the pH value of the environment. Kierczak et al. [22] found that porous GGBS released more trace elements under surface weathering conditions. Ash et al. [23] found that compared with deionized water, rainwater could leach more heavy metals from silver smelting slag over time, and the unevenness of the slag and the contact time were the main factors contributing to the release of toxic metals.

These research results have greatly contributed to the understanding of the material development, macromechanical behaviors, microstructure and environmental impacts of CPB. But no research on the preparation of CPB paste using OSR, SS and GGBS as constituent materials has been reported. Moreover, the micro hydration mechanism and the impact of this new CPB paste on the groundwater environment are unknown.

In this paper, OSR, SS and GGBS are used as constituent materials to prepare a new environmentally friendly COSGB. The response surface method was used to optimize the mix ratio for COSGB. The specimens with the optimum mix ratio were also analyzed for their hydration products, micromorphology and pore structure. Contaminants in COSGB were studied by leaching tests.

## 2. Materials and Methods

### 2.1. Materials

**Figure 1:**
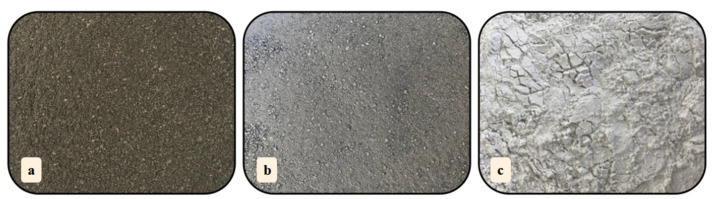
Photos before consolidation: (**a**) OSR, (**b**) SS and (**c**) GGBS.

The OSR in the mixture was taken from the Fushun West Open-pit Coal Mine (123°04′48″–124°27′26″ E, 41°27′10″–42°01′01″ N). This material is solid waste associated with coal and used after crushing. The SS in the experiment was taken from the Anshan Iron and Steel Group (Anshan, China). The GGBS is S95 grade slag produced by Shandong Kangjing New Material Technology Co., Ltd. (Jinan, China). Tap water was used for mixing. Photographs of the OSR, SS and GGBS raw materials are shown in Figure 1.

### 2.2. Specimen Preparation

First, OSR, SS and GGBS were poured into a mixer and stirred for 3 min to ensure that the materials in the mixer were homogeneous. After that, pre-weighed water was poured into the mortar mixer and mixed for 4 min. The slump test was performed immediately after mixing. The COSGB mixture was poured into cylindrical molds with dimensions of Φ50 × 100 mm^2^, and the mold was removed after 24 h of curing [24]. The COSGB specimens were stored under standard curing conditions at a temperature of 20 ± 5 °C and humidity above 96% [25]. These curing conditions were provided by a SHBY-90B curing box.

### 2.3. Mix Design

#### 2.3.1. Design of Single-Factor Experiments

COSGB is a mixture of OSR, SS, GGBS and water. The solid mass concentration is a factor used to determine the mass percentages of solids and liquids. GGBS mixing amount is considered separately as a factor due to its relatively high cost. The mass ratio of SS to OSR is used as a factor because only the qualities of SS and OSR are uncertain. Thus, three factors, GGBS mixing amount, mass ratio of SS to OSR and solid mass concentration, were selected for the mix design. Based on a large number of preliminary experiments, single-factor analysis was performed, and the appropriate ranges for the three factors were determined in turn. The design of single-factor experiments is shown in Table 1. On the basis of single-factor experiment results, the response surface method was used to optimize the mix ratio, using 28 days UCS, slump and cost as three response values.

#### 2.3.2. Cost Calculation

GGBS cost is the purchase price from Shandong Kangjing New Material Technology Co., Ltd., China. As solid wastes, OSR and SS only calculate the processing cost. The unit prices of raw materials are shown in Table 2. The apparent density of the COSGB mixture in the fresh state was measured with the method of the ASTM standard (ASTM C 138) [26]. Then the unit weight of water (kg/m^3^) and three solid materials (kg/m^3^) was calculated according to the mix ratio. Finally, the unit cost of COSGB (USD/m^3^) was calculated based on the unit prices of raw materials.

#### 2.3.3. Multi-Objective Optimization

Response surface methodology (RSM) is an optimization method that integrates experimental design and mathematical modelling. Based on the BBD test results, a response surface model was developed [27]. The form is as follows:

(1) $$y=a_{0}+\overset{k}{\underset{i=1}{\sum }}a_{i}x_{i}+\overset{k}{\underset{i=1}{\sum }}a_{\mathrm{ii}}x_{i}^{2}+\overset{k}{\underset{i=1}{\sum }}\overset{k}{\underset{j=1}{\sum }}a_{\mathrm{ij}}x_{i}x_{j}$$

In Equation (1), *x*_i_ and *x*_j_ represent the independent variables, y represents the design response value, and *a*_0_, *a*_i_, *a*_ii_, and *a*_ij_ are the constant, linear, quadratic, and interaction coefficients.

To obtain the optimal mix ratio of COSGB materials, the desirability function method was used [27].

First, we established a single-response desirability function, *d*_i_.

(2) $$d_{i}=\{\begin{array}{c} 0,\\ {\left[\frac{Y_{i}-L_{i}}{U_{i}-L_{i}}\right]}^{wt_{i}},\\ 1, \end{array}\begin{array}{c} Y_{i}\leq L_{i}\\ L_{i}<Y_{i}<U_{i}\\ Y_{i}\geq U_{i} \end{array}$$

(3) $$d_{i}=\{\begin{array}{c} 0,\\ {\left[\frac{Y_{i}-L_{i}}{T_{i}-L_{i}}\right]}^{wt_{i}},\\ \begin{array}{c} 1,\\ \begin{array}{c} {\left[\frac{U_{i}-Y_{i}}{U_{i}-T_{i}}\right]}^{wt_{i}},\\ 0, \end{array} \end{array} \end{array}\begin{array}{c} Y_{i}\leq L_{i}\\ L_{i}<Y_{i}<T_{i}\\ \begin{array}{c} Y_{i}=T_{i}\\ \begin{array}{c} T_{i}<Y_{i}<U_{i}\\ Y_{i}\geq U_{i} \end{array} \end{array} \end{array}$$

(4) $$d_{i}=\{\begin{array}{c} 1,\\ {\left[\frac{U_{i}-Y_{i}}{U_{i}-L_{i}}\right]}^{wt_{i}},\\ 0, \end{array}\begin{array}{c} Y_{i}\leq L_{i}\\ L_{i}<Y_{i}<U_{i}\\ Y_{i}\geq U_{i} \end{array}$$

In Equations (2)–(4), *d*_i_ denotes the desirability function of the *i*-th response. *Y*_i_ denotes the *i*-th response. *L*_i_ is the lower limit of the *i*-th response value. *U*_i_ is the upper limit of the *i*-th response value. *T*_i_ is the target value of the *i*-th response. Equation (2) is applicable to the response variable in which desirability increases with the increase in the response value. Equation (3) is applicable to the response variable with an optimal target value, and the desirability closest to the set target value is greater; Equation (4) is applicable to the response variable in which desirability increases with the decrease in the response value.

Second, the geometric mean of all responding single expected target *d*_i_ values is the overall desirability function *D* (Equation (5)). ∑*s*_i_ = 1 in Equation (5), the higher *s*_i_ value means that the target value is more important.

(5) $$D={\left(\overset{n}{\underset{i=1}{\prod }}d_{i}^{s_{i}}\right)}^{\frac{1}{\sum s_{i}}}$$

Using a single-response function as a constraint, nonlinear regression was performed for the overall desirability function *D*. The optimal mix ratio was selected for the highest *D* value.

(6) $$ARD=\frac{Exp-Pre}{Exp}\times 100\%$$

Finally, Equation (6) was used to calculate the absolute relative deviation (*ARD*) [28] between the model predictions (*Pre*) and the design experimental (*Exp*) values. The *ARD* reflects the accuracy of response surface model predictions. When the *ARD* is less than 5%, it indicates that the prediction accuracy is higher and the reference ability is greater.

### 2.4. Test Methods

#### 2.4.1. Physical and Mechanical Property Tests

The UCS of the COSGB was tested according to the ASTM C39/C39M-15a [29] specification for uniaxial compression. The testing equipment was a WDW-300 universal testing machine. The slump was tested with a slump cylinder according to the ISO1920-2 [30] specification.

#### 2.4.2. Microscopic Tests

A XRD-6100 X-ray diffractometer (Shimadzu, Kyoto, Japan) was used for phase analysis of the specimens. A JSM-7500F scanning electron microscope (JEOL, Tokyo, Japan) was used to analyse the surface morphology changes. A FYFS-2002E energy dispersive spectroscopy (EDS) detector (Fangyuan Instrument, Wuhan, China) was used for the elemental analysis of hydration products. An IRPrestige-21 (Shimadzu, Kyoto, Japan) Fourier transform infrared (FTIR) spectrometer was used to characterize the molecular structure and chemical bonds of the specimens. An Autopore IV 9500 (Norcross, GA, USA) mercury porosimeter was used to measure the total porosity and pore size distribution of the specimens.

#### 2.4.3. Leaching Experiment

The toxic leaching of the backfill materials was analysed by the horizontal oscillation method (Chinese Standard HJ 557-2010, HJ/T 299-2007) [31,32]. First, filled paste specimens at different curing ages were crushed. After passing through a 3.0 mm sieve (OSR, SS and GGBS particles meeting the size requirements can be directly used), 100 g crushed specimens were weighed and placed in a 2 L extraction bottle. Deionized water (pH = 7.1) or a mixture of concentrated sulfuric acid and concentrated nitric acid (pH = 3.20 ± 0.05) at a liquid to solid ratio of 10:1 (L/kg) was added as the leaching agent. The bottle was fixed on a two-speed thermostatic horizontal oscillator (HZ-9811K, Jiangsu Taicang Science and Education Equipment Factory, Nanjing, China). The oscillation frequency was 110 ± 10 times/min, and after 8 h of shaking at room temperature, the extraction bottles were removed. After 16 h of rest and filtering under pressure, the leachate was collected. The heavy metal ion concentration in the leachate was determined by atomic spectrophotometry (Hitachi, Z-2000, Hitachi, Tokyo, Japan). The pH of the leachate was determined by a pH meter (Remagnetics, PHS-3, Shanghai, China).

## 3. Results and Discussion

### 3.1. Raw Materials Characterization

**Figure 2:**
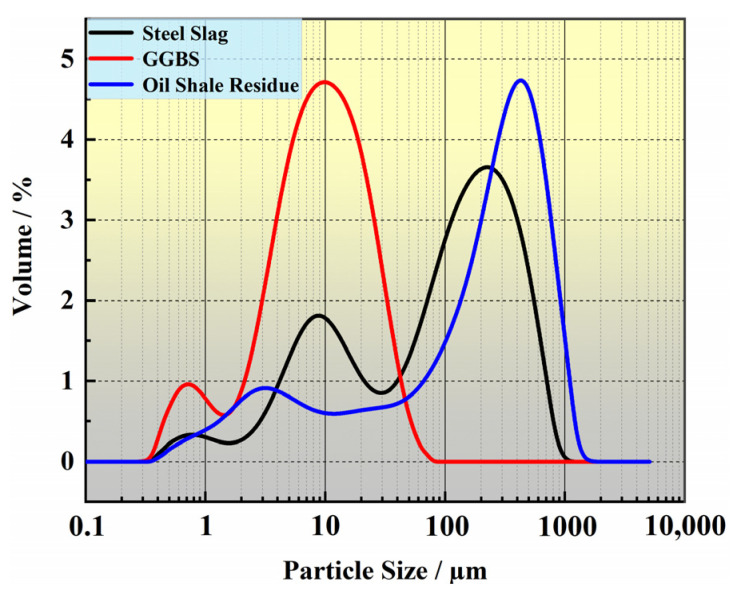
Particle size curves of the OSR, SS and GGBS.

**Figure 3:**
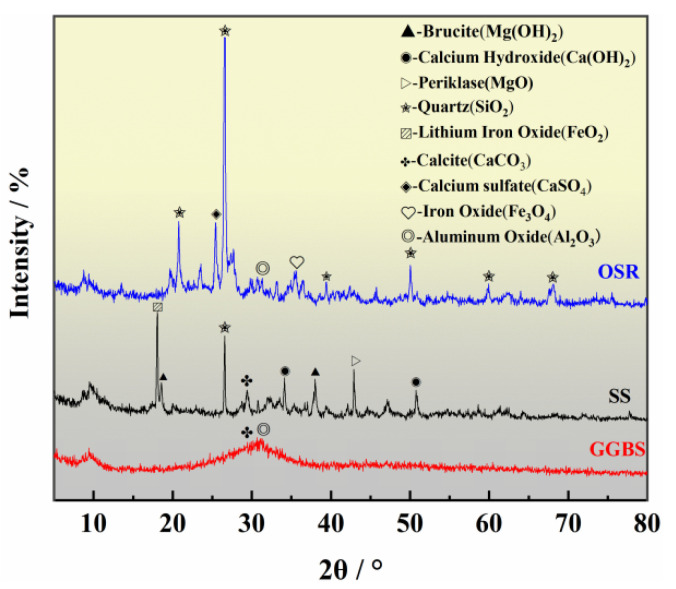
XRD patterns of the OSR, SS and GGBS.

**Figure 4:**
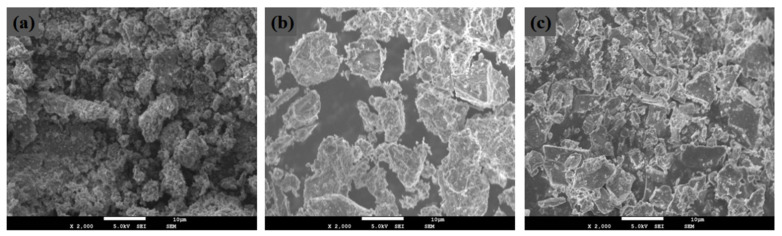
SEM images of the (**a**) OSR, (**b**) SS and (**c**) GGBS.

The OSR is brown, with a particle size is mostly concentrated between 0.6–1 mm, and it is irregular and angular in shape, with a specific surface area of 549 m^2^/kg. The SS is light grey, it is mainly composed of particles and powders with a particle size of less than 1.20 mm, and it has a specific surface area of 519 m^2^/kg. The GGBS is a milky white powder with a uniform particle size of 0.01 mm and a specific surface area of 1570 m^2^/kg. The particle size curves of the three raw materials are shown in Figure 2. The mineral compositions of the OSR, SS and GGBS determined by XRD analysis are shown in Figure 3. The micromorphologies of the three raw materials obtained through SEM testing are shown in Figure 4. The main chemical compositions obtained by X-ray fluorescence (XRF) analysis are shown in Table 3.

### 3.2. Analysis of the Single-Factor Test Results

**Figure 5:**
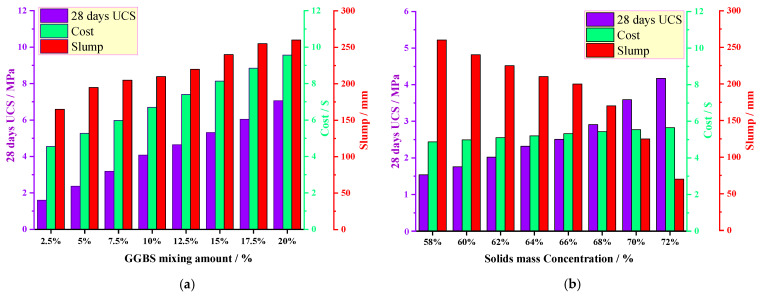

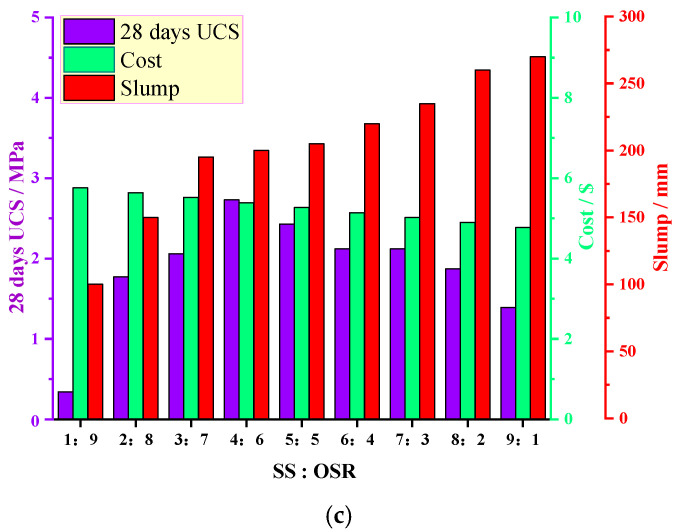
Single-factor effect of the (**a**) GGBS mixing amount; (**b**) solid mass concentration; and (**c**) SS to OSR mass ratio.

The mass ratio of SS to OSR was fixed at 1:1, and the solid mass concentration was 65%. The effects of the GGBS mixing amount on the 28 days UCS, slump and cost of the COSGB were obtained by varying the GGBS mixing amount, as shown in Figure 5a. From Figure 5a, the 28 days UCS of the COSGB shows an obvious growth trend with increasing GGBS mixing amount, and the slump increases with increasing GGBS dose. Additionally, with increasing GGBS, the fluidity is enhanced, the water secretion rate increases, and the cost increases. Considering these factors, the range of the GGBS mixing amount was set at 2.5~7.5%. At this time, the 28 days UCS of the COSGB meets the requirements of CPB, the cost is relatively low, and the fluidity is relatively good.

When the mass ratio of SS to OSR was fixed at 1:1 and the GGBS mixing amount was 5%, the solid mass concentration was adjusted, and the effects of the solid mass concentration on the UCS, slump and cost of the COSGB were obtained, as shown in Figure 5b. Figure 5b shows that the 28 days UCS of the COSGB increases with increasing solid mass concentration. Additionally, the cost factor is relatively minimally influenced by the solid mass concentration. The slump tends to decrease gradually with increasing solid mass concentration of the COSGB. The range of the solid mass concentration was set at 64~68% in combination with the requirements of the liquidity of CPB.

The effects of the SS to OSR mass ratio on the 28 days UCS, slump and cost of the COSGB were obtained by adjusting the SS to OSR mass ratio at a solid mass concentration 65% and GGBS mixing amount 5%, as shown in Figure 5c. Figure 5c shows that the 28 days UCS of the COSGB tends to increase and then decrease with an increasing mix ratio of SS to OSR, and the slump increases with increasing mix ratio. Therefore, the influence of the SS and OSR mixing amount on the flowability of the COSGB is extremely significant. Since both OSR and SS are waste materials, the SS to OSR mass ratio does not have a large impact on the cost factor. From the Figure 5c, the 28 days UCS and flowability are good when the ratio of SS to OSR is between 3:7 and 5:5. The ratio of SS to OSR was set between 3:7 and 5:5 (recorded as 0.4~1).

### 3.3. Analysis of the Response Surface Method Results

#### 3.3.1. Design Scheme of the BBD

Based on the analysis results of the above single-factor analysis, Table 4 shows the mix ratio data determined by the Box–Behnken (BBD) response surface design.

#### 3.3.2. Experimental Results of the BBD

Based on the BBD method, 17 sets of mix ratio (including 5 center point replicates) were designed and carried out, and 28 days UCS and slump tests as well as cost calculations were performed for different mix ratio. The mix ratios and response values are shown in Table 5.

#### 3.3.3. Response Surface Model Fitting and Validation

According to the test results obtained in Table 5, the response surface function was fitted using the second-order model in Equation (1).

The formula used to fit response 1 (28 days UCS) is shown in Equation (7):

*Y*_1_ = 43.18 − 0.82*A* − 2.08*B* − 1.38*C* − 0.12*AB* + 0.02*AC* + 0.02*BC* − 0.02*A*^2^ + 0.58*B*^2^ + 0.01*C*^2^ (*R*^2^ = 0.9875) (7)

The formula used to fit response 2 (slump) is shown in Equation (8):

*Y*_2_ = 5771.87 − 20.42*A* − 1833.33*B* − 136.98*C* − 3.33*AB* + 0.25*AC* + 29.17*BC* + 0.90*A*^2^ + 20.83*B*^2^ + 0.78*C*^2^ (*R*^2^ = 0.9834) (8)

The formula used to fit response 3 (cost) is shown in Equation (9):

*Y*_3_ = 183.47 − 0.28*A* − 2.08*B* − 5.44*C* + 3.33 × 10^−3^*AB* + 4.50 × 10^−3^*AC* − 0.02*BC* + 0.03*A*^2^ + 2.13*B*^2^ + 0.04*C*^2^ (*R*^2^ = 0.9994) (9)

The results of the analysis of variance (ANOVA) obtained by conducting significance tests are shown in Table 6.

For each significant factor in the model, the *p*-value is the main consideration. If *p* < 0.05, the factor is significant in the model. Otherwise, it is not significant. When *p* < 0.01, the factor is highly significant in the model [33]. The *p*-values of the regression models for *Y*_1_, *Y*_2_ and *Y*_3_ were all less than 0.01, indicating that these mathematical models were statistically significant. Moreover, the *R*^2^ values of the correlation coefficients for each model fit were above 0.90. *Y*_1_, *Y*_2_ and *Y*_3_ were 0.9875, 0.9834 and 0.9994, respectively. The closer the value of the correlation coefficient (*R*^2^) was to 1, the more accurate the fit was. The above results fully show that the measured values of compressive strength, slump and cost of the COSGB are in good agreement with the predicted values. Thus, Equations (7)–(9) fit the experimental results well and are able to accurately predict the 28 days UCS, slump and cost within the given range.

#### 3.3.4. Analysis of the Response Surface Interaction Impact

The 3D response surface provides a more intuitive description of the relationship between the interaction of the two factors and the response value, so that the effect of changes in the levels of the factors on the response value can be generalized. The greater the curvature of the response surface is, the more significant the effect of factor interactions is. Conversely, the impact of the factor is not significant [34].

**Figure 6:**
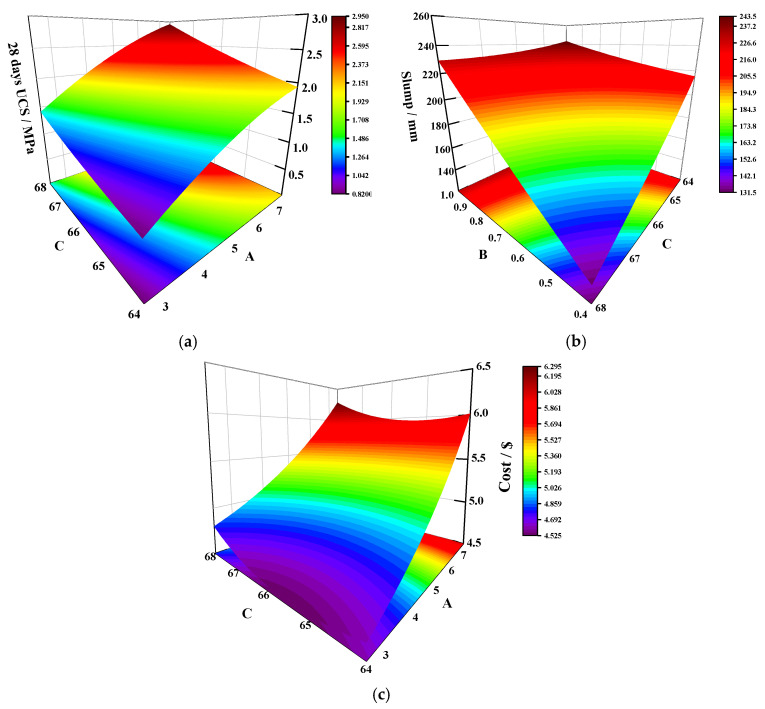
Interaction response surface plots of factors: (**a**) effect of factor *AC* on the 28 days UCS; (**b**) effect of factor *BC* on the slump; and (**c**) effect of factor *AC* on the cost.

Based on the experimental results and the variance analysis of the 28 d UCS, the *P*-value for *AC* is 0.0464 (<0.05), indicating that the interaction between *AC* is more obvious than the other interactions. The three-dimensional response surface for the *AC* interaction is shown in Figure 6a. As the decrease of *A* and *C*, the curvature of the 28 days UCS response surface (28 days UCS growth rate) increases. This suggests that the decrease in total solids is accompanied by an attenuation of the 28 days UCS. The increase in total solids means a decrease in the total water in the COSGB. On the one hand, a reduction in water leads to a lower initial porosity of COSGB and tighter bonding of the COSGB matrix. On the other hand, the lower water content in the COSGB indirectly enhances the alkali concentration in the contact environment and promotes the hydration process of the mixture.

For response 2 (slump), as can be seen in the ANOVA results (Table 6), *p* < 0.01 for *BC* indicates the highly significant interaction of *BC*. The 3D response surface of the *BC* interaction is shown in Figure 6b. From Figure 6b, the 3D response surface is clearly curved, indicating a significant interaction between the *B* and *C* factors. As *B* decreases and *C* increases, the curvature of the slump response surface (the growth rate of the slump) increases. The increase in the ratio of SS to OSR significantly increases the slump value, because OSR is composed of loose, irregularly shaped and angular fine particles with very rough and water-absorbent surfaces. The high friction between the mixture and SS particles reduces the fluidity of the COSGB mixture, resulting in a lower slump value of COSGB mixture. In addition, the low density of OSR results in a large amount of OSR at the same mass, which is also an important factor affecting the solid mass concentration. The increase in OSR indirectly affects the slump of the COSGB mixture.

Regarding the response values of cost, the *p*-values obtained for the interaction terms of *BC* and *AC* are less than 0.05, showing that they all have a significant impact on the cost. The *AC* interaction with a relatively smaller *p*-value is given as an example in Figure 6c. GGBS is known to be the main cost source in COSGB without any chemical additives. Thus, the main source of interaction effects is the value of the COSGB material itself. Under the same conditions, the solid mass concentration increases from 64% to 68%, and the cost increases from 4.62 USD to 4.80 USD, which is an increase of 3.90%. This indicates that an increase in the solid mass concentration is accompanied by an increase in cost, but the increase extent is small. This conclusion is consistent with the impact of a single-factor on the cost.

#### 3.3.5. Response Surface Multi-Objective Optimization

The COSGB is designed to maximize the strength of the backfill and minimizing the cost per cubic meter of COSGB material, while meeting the flow requirements needed for backfill. Therefore, the individual desirability functions used for the COSGB optimization are set as follows: the maximum value is chosen for the 28 days UCS (Equation (2)), the slump target is 200 mm [12] (Equation (3)), and the minimum value is chosen for the cost (Equation (4)). The weighting factor *w*_ti_ = 1 is chosen in this study. In this study, the 28 days UCS is selected and has the same importance as slump and cost [35] (*s*_1_ = *s*_2_ = *s*_3_ = 1/3).

Based on using a single-response function as a constraint, nonlinear regression was performed for the overall desirability function *D* (Equation (5)). The optimal mixture ratio was selected for the highest *D* value. The final results are as follows: the GGBS mixing amount is 4.72%, the mix ratio of SS to the OSR is 0.82, and the solid mass concentration is 67.69%. And the predicted (*Pre*) response values are as follows: 28days UCS of 2.10 MPa; slump of 200 mm; cost of 5.21 USD. The experimental (*Exp*) values of COSGB obtained by the optimal mixture ratio test are as follows: 28 days UCS of 2.12 MPa; slump of 205 mm; cost of 5.17 USD.

According to Equation (6), the *ARD*s for the 28 days UCS, slump and cost are 0.94%, 2.44% and −0.77%, respectively. All of the errors are less than 5%, which indicates that the prediction accuracy is high and the prediction has a strong reference value for the optimization results of COSGB.

**Figure 7:**
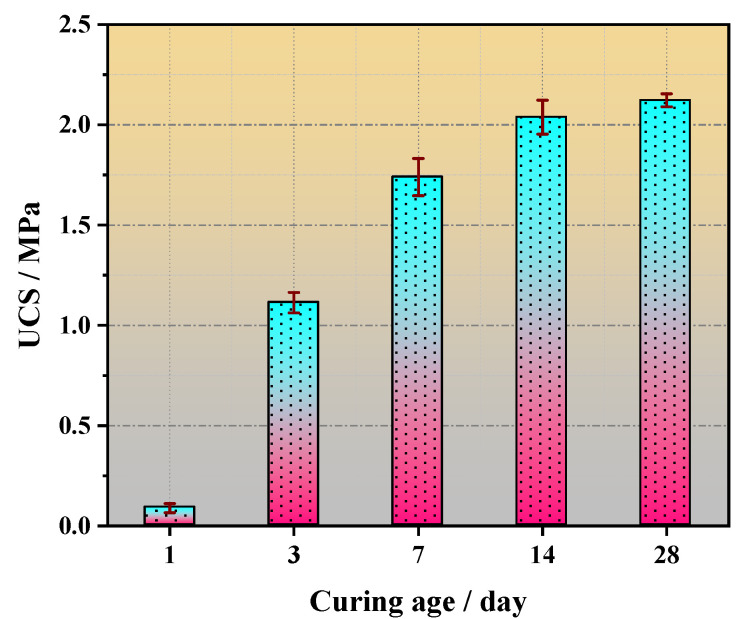
Variation in strength with curing age for the optimal mixture ratio of COSGB.

In the meantime, the variation in the strength of the COSGB with curing age for the optimal mix ratio is shown in Figure 7.

The change in the UCS of COSGB with the curing age is shown in Figure 7. According to Figure 7, the UCS of COSGB increases with increasing curing age. The 7 days and 28 days UCS are 1.74 MPa and 2.12 MPa, respectively, and the 7 days UCS is 82% of the 28 days UCS. However, it could be concluded from the references [8,12] that the 7 days UCS of the traditional cement-based material was only 60–70% of the 28 days UCS. This shows that the developed COSGB is an early-strength backfill, and filling goaf with COSGB can quickly strengthen and support roofs to ensure the smooth progress of mining filling. In addition, traditional CPB uses OPC as a binder, and the production of OPC will be accompanied by a large amount of CO_2_ emissions. While OSR, SS and GGBS used in COSGB are all solid wastes. Therefore, COSGB is more environmentally friendly than traditional CPB.

### 3.4. Microstructural Analysis of COSGB

The optimal COSGB mix ratio was used. At different curing ages, the hydration mechanism of the three raw materials was analyzed with a combination of XRD, FTIR, SEM-EDS and MIP.

#### 3.4.1. XRD Analysis

**Figure 8:**
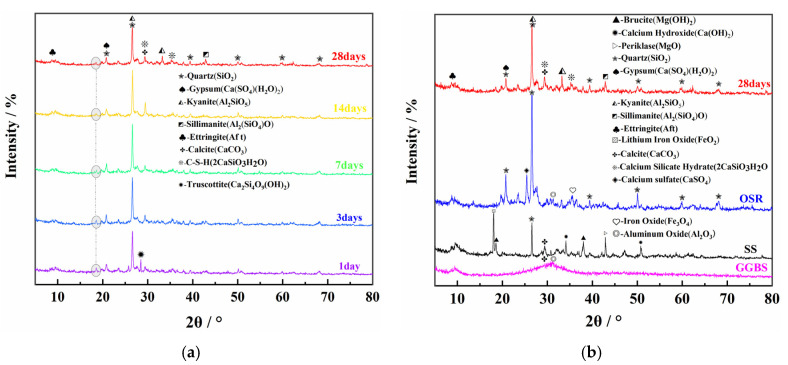
XRD patterns of COSGB before and after reaction: (**a**) COSGB at different curing ages and (**b**) OSR, SS and GGBS at curing ages of 28 d.

The XRD patterns of OSR, SS, GGBS and COSGB at different curing ages are shown in Figure 8.

As shown in Figure 8a, the XRD patterns of the specimen at each curing age exhibit more distinct fluctuating peaks in the 2*θ* range of 20–45°, which indicates the coexistence of amorphous silicate gels [36]. Crystalline phases of calcite (CaCO_3_) and quartz (SiO_2_) are present in the COSGB after mixing and curing. These phases are derived from unreacted SS and OSR particles. Under longer curing ages, internal kyanite (Al_2_SiO_5_), a type of mullite, is exposed with the dissolution of the surface crust of OSR particles [37]. In the XRD patterns, characteristic peaks of ettringite (AFt), hard gypsum (Ca(SO_4_)(H_2_O)_2_) and calcium silicate hydrate (C-S-H) are detected in the cured matrix.

As shown in Figure 8b, the XRD patterns of COSGB with curing age of 28 days are compared with those of OSR, SS and GGBS before mixed curing. From Figure 8b, the crystalline peaks, including calcium sulfate (CaSO_4_) and ferric oxide (Fe_3_O_4_) that are originally present in OSR and the magnesium hydroxide (Mg(OH)_2_) and calcium hydroxide (Ca(OH)_2_) that are originally present in SS, disappeared [38]. This shows that these components dissolve due to the alkali activation reaction after mixing. Because OSR used is strongly alkaline, the Ca(OH)_2_ produced by hydrolysis and the Ca(OH)_2_ in SS make the hydration environment gradually alkaline. This creates conditions for the dispersion and dissolution of GGBS vitreous, and promotes the continuous hydration reaction of SiO_2_ and A1_2_O_3_ in the mixture. Additionally, the Al_2_O_3_ and SiO_2_ presenting in OSR and GGBS, and the Ca(OH)_2_ of SS, can generate AFt with CaSO_4_ by hydration reaction. AFt is one of the sources enhancing the UCS of the COSGB [39]. The mixing of minerals consumes Ca(OH)_2_ to form C-S-H gels [40]. Therefore, it is hypothesized that C-S-H gels and calcium alumina produced by excitation of shale slag on steel slag and mine slag may be the main source of strength of COSGB materials. This conclusion was verified by SEM-EDS analysis of the hydration products.

It should be noted that there are two more special changes in Figure 8a. One change was that trublite (Ca_2_Si_4_O_9_(OH)_2_) only appeared at the 1d curing age, and it was not found during the rest of the curing ages. Because the chemical composition and short-term structure of C-S-H gels are similar to those of natural zeolites. C-S-H gels with zeolite-like structures may appear at short curing ages. This is consistent with the conclusion of Hanjitsuwan et al. [41]. The other change was that the special diffraction peak gradually faded in 1–7 days. The peak is the same as peak of the PDF card of the calcium alumino silicate hydrate (C-A-S-H). Due to the relatively low contents of Al in the raw materials, it can be judged that C-A-S-H gels exist in small amounts at the early stage. C-A-S-H gel is encapsulated by C-S-H gel with the increase of curing age. Therefore, the crystallinity is so poor that the diffraction peaks gradually decrease and become stable. Yang et al. [42] also found similar phenomenon by analyzing the hydration products of alkali-activated materials. In this regard, FTIR analysis for special waveform shifts has also led to the same conclusion.

#### 3.4.2. FTIR Analysis

**Figure 9:**
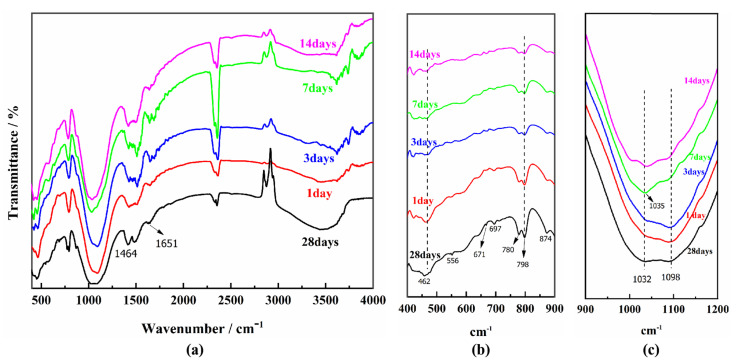
FTIR spectra of COSGB at different curing ages: (**a**) complete spectra; (**b**) enlargement of the 400–900 cm^−1^; (**c**) enlargement of the 900–1200 cm^−1^.

The FTIR spectra of the COSGB at different curing ages are given in Figure 9a to identify the hydration gel products of material. Figure 9b,c are enlarged images of the interval.

As shown in Figure 9a, the presence of quartz in COSGB causes the FTIR spectrum to rise to a range of bands located at 1035, 1098, 780–798 (double bands), 697 and 556 cm^−1^ [43]. A series of bands at 1030–1130 cm^−^^1^ and 550–560 cm^−^^1^ are related to the presence of mullite (bands associated with the presence of octahedral aluminum in mullite). This is consistent with the results obtained from the XRD analysis [44]. The peak at 1651 cm^−1^ is the vibrational band of –OH and H_2_O, and the peak at 1464 cm^−1^ is the vibrational band of –OCO–, representing the formation of carbonates. Combined with XRD analysis, it can be concluded that the product is CaCO_3_.

The peaks at 550 and 870 cm^−^^1^ correspond to Al–O and Ca–O vibrational bands respectively, and the absorption peak of crystalline water appears at 1651 cm^−^^1^, which indicate the formation of AFt during hydration action. From the curves in Figure 9a, the region between 900 and 1200 cm^−1^ overlaps due to the asymmetric stretching vibration of Si–O–T (Si or Al) in the C (A)-S-H gels. The vibration results in the produce of a broad and intense band in the abovementioned interval [45]. Additionally, the deformation vibration appears inside the T–O bond at 462 cm^−^^1^ in Figure 9b. These indicate that the COSGB after the reaction has a high degree of structural heterogeneity and forms C-S-H gels. The research results of many scholars support this conclusion [46,47].

It is noteworthy from Figure 9c that the Si–O–T (Si or Al) asymmetric stretching vibrational band shifts to lower wavenumbers (from 1098 cm^−1^ to 1032 cm^−^^1^) with increasing curing age. Criado et al. [48] suggest that this situation arises due to the successive formation of two different gels. One is C-A-S-H gels generated in early stage, the other is C-S-H gels gradually evolved from C-A-S-H gels (mainly enhancing the mechanical strength of the COSGB). This corresponds to the analytical results of the disappearance of the characteristic peaks of the C-A-S-H gels in the XRD analysis. So, the UCS increases rapidly within the 1–7 days curing ages. After curing 7 d, the UCS reaches 82% of 28 days UCS. This is why COSGB materials have early strength properties.

In summary, the main hydration products of the COSGB are a mixture of AFt and C-S-H gel, which is consistent with the XRD analysis.

#### 3.4.3. SEM-EDS Analysis

**Figure 10:**
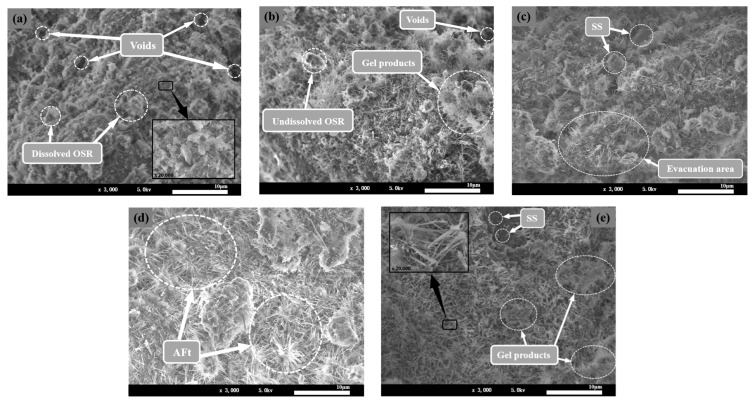
SEM images of COSGB at curing ages of 1 day (**a**), 3 days (**b**), 7 days (**c**), 14 days (**d**) and 28 days €.

**Figure 11:**
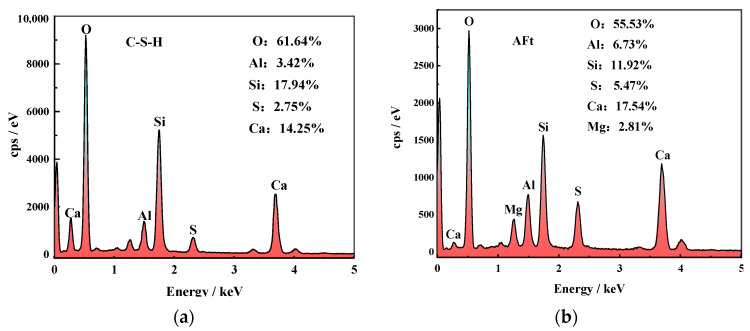
EDS energy spectra: (**a**) C-S-H gel and (**b**) AFt.

To investigate the microstructure of COSGB, SEM and EDS analyses were performed on specimens with different curing ages (Figure 10 and Figure 11).

The internal structure and the changes in the hydration reaction products of the COSGB were characterized by SEM-EDS. As shown in Figure 10, the microstructure of the COSGB specimens becomes denser with increasing curing age. The microstructures of the specimens at 1 day, 3 days and 7 days curing ages show relatively sparse, porous and inhomogeneous morphologies. Many undissolved solid particles and voids are observed, indicating a low reaction degree of alkaline OSR to excite GGBS and SS. At 14 days and 28 days, the microstructure is denser and more homogeneous, and more needle-like formations in the matrix can be observed. The EDS spectrum in Figure 11b shows that the needle-like product is AFt. In Figure 11a, the EDS spectrum of the flocculent has high contents of elemental Ca and Si, and thus, the flocculent is determined to be a C-S-H gel [49]. This is consistent with the results of the XRD and FTIR analyses described previously.

At 1 day curing age, a C-(A)-S-H gel forms at the beginning of the hydration reaction after mixing. Then more gel products form, not only filling the existing voids but also binding the remaining solid particles together. As shown in Figure 10a, the feedstock uniformly fills the entire space of the COSGB, with undissolved SS and OSR particles randomly scattering throughout the interior of the COSGB. Additionally, the SS particles are coarser than those of the remaining two materials and participate in the cementation process as fine aggregates [39]. Thus, a continuous, dense and complete matrix forms.

Figure 10c,e shows that the AFt in the sparsely distributed mixture grows uniformly, and thus, the effective particle gradation increases the contact area among the particles and promotes the occurrence of hydration reactions, which in turn reduces the porosity and increases the compressive strength at the same time. By enlarging the needles in Figure 10a,e at the same magnification (×20,000), we find that the amount and volume of AFt increase as the curing age increases. Both C-S-H gel and AFt fill the original particle size gap to form a denser structure, increasing the UCS of the COSGB at longer curing age. Many scholars have found similar conclusions in studies of cement and concrete [50,51].

#### 3.4.4. MIP Analysis

**Figure 12:**
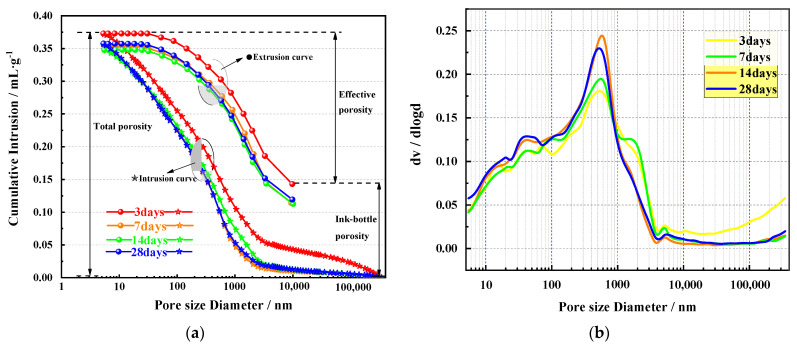
MIP results obtained for specimens at different curing ages: (**a**) total porosity and effective porosity; (**b**) pore size distribution.

The test results of the pore structure of the specimens at different curing ages are analyzed using the MIP test method (as shown in Figure 12). The MIP results of the total porosity and effective porosity of COSGB specimens measured at 3 days, 7 days, 14 days and 28 days are shown in Figure 12a. Figure 12b shows the pore size distribution of the COSGB specimens measured at different curing ages.

The MIP test results show that the cumulative pore volume (CPV) increases with decreasing pore diameter. During the test, continuous pores in the COSGB specimen, including “ink-bottle” pores, can be detected from the intrusion curves [52]. The total porosity minus the “ink-bottle” porosity is the “effective porosity”. It can be concluded that the overall variation in effective porosity is not significant [53]. Combined with Figure 12b, the number of less harmful pores (20–50 nm) and harmful pores (50–200 nm) increase slightly with age, accompanied by a relative decrease in the number of more harmful pores (>200 nm). This is consistent with the reduction in the number of pores observed by SEM. In addition, Table 7 lists the total pore areas, median pore diameters and porosities of the COSGB specimens.

Combined with the graphs, the total pore area, median pore diameter and porosity of COSGB specimens all decrease to varying degrees with increasing curing age. This result verifies that the hydration reaction continues after 3 days, and performed reaction products fill the original pores, promoting the conversion of large pores to small pores. It is worth noting that the same results were observed as for Portland cement paste, where the pore size and porosity generally decreased with hydration [54,55]. It follows that hydration promotes the growth of hydration products filling in the pores. The combination of the XRD, IR, and SEM-EDS results shows that in this material, the generation of C-S-H gels and the growth of AFt lead to a significant decrease in the proportion of sparse and harmful pores and an increase in the proportion of denser and less harmful pores, which helps to improve the axial load bearing capacity of COSGB. Macroscopically, the UCS of the COSGB material gradually increases with curing age. Many studies have also proven the close correlation between the compressive strength and porosity of cementitious materials [15,56].

### 3.5. Analysis of Leaching Experiment Results

Filling materials such as OSR, SS and GGBS may carry heavy metals and other pollutants. Whether the formation of filled paste poses a threat to the groundwater environment needs to be measured and analyzed.

Table 8 shows the results of the leaching experiments performed for the raw backfill materials, including OSR, SS and GGBS. We consider the effects of both neutral and acidic groundwater at the mine site on leaching. The water leaching and acid leaching parameters are set to pH = 7.1 and pH = 3.2, respectively. Table 8 shows that the acid leaching concentration is greater than the water leaching concentration for the leaching of contaminants from the same backfill material. This is one of the reasons for the serious groundwater pollution in acid mine wastewater areas. According to the Chinese groundwater quality standard (GB/T 14848-2017) [57], the Fe^2+^, Mn^2+^ (acid leaching) and Cr^6+^ contents in the leachate of OSR and SS exceed the standard, and the Fe^2+^ content in the leachate of GGBS exceeds the standard. As shown in Table 8, the pH values of all leachates from the three raw materials are alkaline, which is why they can promote the strength of COSGB without alkaline excitation.

**Figure 13:**
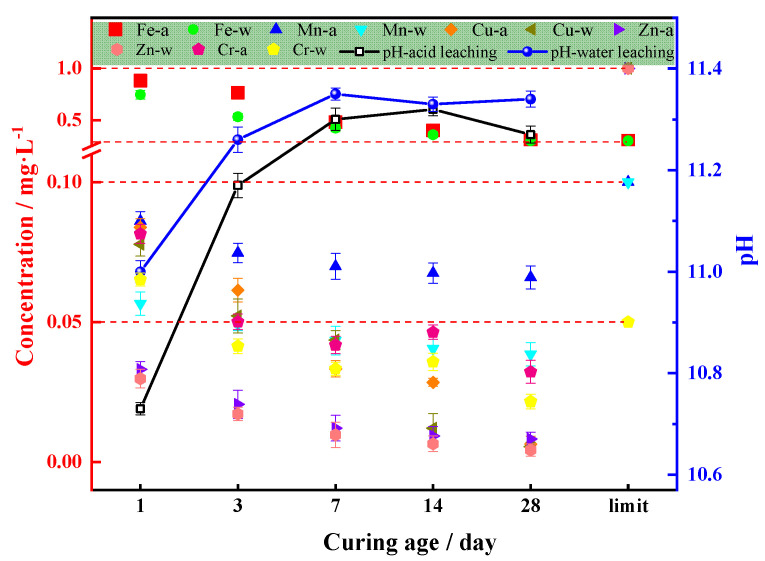
Variation in the COSGB contaminant leaching concentration at different curing ages.

Figure 13 shows the leaching contaminants of specimens, which maintain for 1 day, 3 days, 7 days, 14 days and 28 days and then crush through a 3 mm sieve with the optimal mixture ratios.

The red line in Figure 13 indicates the standard concentration limit of each heavy metal ion according to the Chinese groundwater quality standard. As seen in Figure 13, the leaching levels of the COSGB are lower than the levels defined in the Chinese groundwater quality standard in the water (-w in Figure 13) and acid leaching (-a in Figure 13) states except Fe^2+^, which slightly exceeds the standard. Fe^2+^ content of the original backfill materials, the SS and OSR, are high, which results in the slight overrun of Fe^2+^ leaching after backfilling and mixing. The leaching concentrations of all ions except Fe^2+^ are below 0.1 mg/L. Comparing the water leaching with acid leaching conditions, the leaching concentration of heavy metals is greater under acid leaching conditions than under water leaching conditions, which is consistent with the leaching of raw materials. From the curing age of the COSGB, the concentration of Cr(VI) fluctuates with increasing curing age in the order 1 day > 3 days > 14 days > 7 days > 28 days, and the leaching concentration of other heavy metal ions keeps decreasing. With the increase in curing age, the pore size of the hydrated gel decreases, the permeability decreases, the contaminants are encapsulated, and the migration characteristics are greatly reduced. The pH of the leachate is alkaline and fluctuates between 10.7 and 11.4 as the curing age increases. The alkaline chemical reaction also inhibits the migration of many heavy metals. From another point of view, acid mine water can be neutralized, but this is unfavourable for neutral or alkaline mine water. In general, the concentration of contaminants leached from the developed COSGB meets the requirements of the Chinese groundwater quality standard, and thus, it is safe for application in backfill.

## 4. Conclusions

This study explored the possibility of using CPB, OSR, SS and GGBS without the addition of any chemical reagents as solid waste cementation filling. The optimal mix ratio was found through single-factor analysis and the BBD response surface method. In addition, a variety of microstructural characterization techniques were used to gain insight into the hydration mechanism. The environmental impact of COSGB based on the optimal ratio was evaluated. The main conclusions summarized from the experimental results are as follows.

(1) A new cemented oil shale residue-steel slag-ground granulated blast furnace slag backfill (COSGB) without additives was developed. By optimizing the mix ratio, the GGBS mixing amount is 4.85%, the mass ratio of SS to OSR is 0.82, and the solid mass concentration is 67.69%. For the optimal mix ratio of COSGB, the 28 days UCS value is 2.12 MPa, the slump value is 205 mm, and the cost value is 5.17 USD/m^3^.
(2) As the curing age increases, the connection porosity of the COSGB material decreases significantly, and the UCS gradually increases, reaching 82% of the day 28 strength at 7 days, and slowly increasing in the later period. The alkaline environment is produced by the Ca(OH)_2_ from SS and the hydration products of OSR, which dissolves the GGBS vitreous and promotes the hydration reaction inside the COSGB mixture. At shorter curing age, the hydration products are mainly C-A-S-H gels and C-S-H gels. With increasing curing age, the amount of hydration products increases rapidly. At the same time, the needle-like AFt crystals are combined with the flocculated C-S-H gel to make the COSGB structure more compact and improve the overall stability.
(3) The pH of the leachate of the three raw materials is alkaline. This is why the strength increases without the addition of an alkali exciter to the COSGB material. The leaching concentrations of the heavy metal ions of COSGB meet the requirements of the Chinese groundwater quality standard (GB/T 14848-2017).
(4) Using OSR, SS and GGBS as raw materials, a safe, environmentally friendly and economical COSGB was prepared. The new COSGB material replaces traditional OPC, controls carbon emissions and reduces the cost of CPB materials. The technology has achieved the maximum utilization of industrial solid wastes (OSR, SS and GGBS), and avoided the damage to the ecological environment due to the accumulation of OSR and SS. It is an effective way for cleaner production in the mining industry. When applied to backfill, the leaching process of COSGB cannot cause harm to water environment. This provides a broader application prospects for COSGB materials.
(5) This study focuses on only one area of OSR and SS. In the future, the effects of OSR and SS from different areas on the preparation of COSGB should be further studied. However, due to the complexity, variability, high requirements and harsh implementation environment of the mining industry, the paper does not fully consider the evolution of the mechanical properties of COSGB in complex environments and its impact on the water environment. In the next step, it is necessary to study the influences of environmental factors such as groundwater pH, salt corrosion, temperature and stress conditions on the mechanical properties and toxic leaching of COSGB.

## Figures and Tables

**Table 1:** Design of single-factor experiments.

Constant Factors		Control Factor
Mass ratio of SS to OSR 1:1	Solid mass concentration 65%	GGBS mixing amount
		2.5%, 5%, 7.5%, 10%, 12.5%, 15%, 15.5%, 17.5%, 20%
Mass ratio of SS to OSR 1:1	GGBS mixing amount 5%	Solid mass concentration
		58%, 60%, 62%, 64%, 66%, 68%, 70%, 72%
Solid mass concentration 65%	GGBS mixing amount 5%	Mass ratio of SS to OSR
		1:9, 2:8, 3:7, 4:6 5:5, 6:4, 7:3, 8:2, 9:1

**Table 2:** The unit prices of raw materials.

Material	OSR	SS	GGBS	Water
Unit Price (USD/kg)	0.0038	0.0031	0.0141	0.000665

**Table 3:** Main chemical compositions of raw materials by XRF analysis (wt %).

Material	Al_2_O_3_	CaO	SiO_2_	Fe_2_O_3_	MgO	SO_3_	Na_2_O
OSR	14.71	37.15	31.20	8.63	2.37	0.82	0.04
SS	6.15	40.79	11.24	14.17	21.21	2.79	0.03
GGBS	14.84	44.65	27.62	0.34	6.25	4.11	0.01

**Table 4:** Box–Behnken (BBD) experimental design.

Factors	Code	Unit	Level		
			−1	0	1
GGBS mixing amount	*A*	%	2.5	5	7.5
mix ratio of SS to OSR	*B*	-	0.4	0.7	1
solid mass concentration	*C*	%	64	66	68

**Table 5:** Experimental BBD results.

Test Group	Factor 1	Factor 2	Factor 3	Response 1	Response 2	Response 3
	*A*	*B*	*C*	28 Days UCS	Slump	Cost
	%	-	%	MPa	mm	USD
1	2.5	0.4	66	1.86	160	4.87
2	7.5	0.7	64	2.44	235	6.02
3	7.5	1.0	66	3.38	250	6.05
4	2.5	1.0	66	1.57	235	4.60
5	5.0	0.7	66	2.74	200	5.10
6	2.5	0.7	64	1.29	230	4.62
7	5.0	0.4	68	4.12	135	5.73
8	5.0	0.7	66	2.44	200	5.10
9	5.0	0.7	66	2.57	195	5.10
10	2.5	0.7	68	2.19	185	4.80
11	5.0	0.7	66	2.36	205	5.10
12	5.0	0.4	64	2.4	220	5.47
13	5.0	0.7	66	2.65	200	5.10
14	7.5	0.7	68	3.55	190	6.29
15	5.0	1.0	68	2.8	225	5.42
16	5.0	1.0	64	2.31	240	5.21
17	7.5	0.4	66	4.24	185	6.31

Note: The values of the 28 days UCS and slump in the table are the averages of three measurements.

**Table 6:** ANOVA with the regression model of different response surfaces.

Source	Sum of Squares			Mean Square			*F*-Value			*p*-Value		
	*Y*_1_	*Y*_2_	*Y*_3_	*Y*_1_	*Y*_2_	*Y*_3_	*Y*_1_	*Y*_2_	*Y*_3_	*Y*_1_	*Y*_2_	*Y*_3_
Model	5.40	14406.25	4.87	0.60	1600.69	0.54	61.24	45.97	1514.07	<0.0001	<0.0001	<0.0001
*A*	3.47	378.13	4.18	3.47	378.13	4.18	354.53	10.86	0.000017	<0.0001	0.0132	<0.0001
*B*	0.44	7812.5	0.15	0.44	7812.5	0.15	45.12	224.36	4235	0.0003	<0.0001	<0.0001
*C*	1.29	4753.13	0.11	1.29	4753.13	0.11	131.54	136.5	2962.4	<0.0001	<0.0001	<0.0001
*AB*	0.032	25	0.000025	0.032	25	0.000025	3.31	0.72	0.7	0.1117	0.4248	0.4304
*AC*	0.046	6.25	0.00203	0.046	6.25	0.00203	4.72	0.18	56.7	0.0464	0.6845	0.0001
*BC*	0.0004	1225	0.000625	0.0004	1225	0.000625	0.041	35.18	17.5	0.8456	0.0006	0.0041
*A* ^2^	0.10	133.22	0.12	0.10	133.22	0.12	1043	3.83	3258.5	0.0145	0.0914	<0.0001
*B* ^2^	0.011	14.8	0.15	0.011	14.8	0.15	1.15	0.43	4312.18	0.3188	0.5352	<0.0001
*C* ^2^	0.00824	41.12	0.12	0.00824	41.12	0.12	0.84	1.18	3258.5	0.3894	0.3132	<0.0001
Residual	0.069	243.75	0.00025	0.00979	34.82	0.000035	-	-	-	-	-	-
*Pure Error*	0.014	50	0	0.00343	12.5	0	-	-	-	-	-	-

**Table 7:** Main pore structure parameters of the tested COSGB specimens.

Curing Age	Total Intrusion Volume (mL/g)	Total Pore Area (m^2^/g)	Median Pore Diameter (Volume) (nm)	Median Pore Diameter (Area) (nm)	Average Pore Diameter (nm)	Porosity (%)
3 days	0.3720	28.022	345.53	14.93	59.47	51.45
7 days	0.3523	26.695	270.78	14.91	56.76	48.99
14 days	0.3482	24.539	228.11	14.65	52.79	48.37
28 days	0.3568	23.297	224.04	14.25	50.43	47.76

**Table 8:** Leaching results obtained for OSR, SS and GGBS.

Material	pH	Fe		Mn		Cu		Zn		Cr	
		pH = 7.1	pH = 3.2	pH = 7.1	pH = 3.2	pH = 7.1	pH = 3.2	pH = 7.1	pH = 3.2	pH = 7.1	pH = 3.2
OSR	12.25	0.5082	0.9349	0.0778	0.1062	0.0121	0.134	0.0292	0.0424	0.0593	0.0857
SS	10.44	1.0066	1.4987	0.0968	0.137	0.0072	0.0085	0.0225	0.0341	0.0927	0.1531
GGBS	9.85	0.3128	0.3693	0.0491	0.0713	0.0078	0.0096	0.0172	0.0265	ND	ND
Standard limits	6.5–8.5	0.3		0.1		1.0		1.0		0.05	

Note: (1) ‘ND’ in the table represents ‘not detected’ because the concentration is below the detection limit of the method. (2) All values in the table are the average of three measurements.

## Data Availability

Data can be obtained from corresponding authors upon reasonable request.

## Abbreviations

AASs	acidic blast furnace slags
ANOVA	analysis of variance
ARD	absolute relative deviation
BBD	Box–Behnken Design
CASH	Calcium alumino silicate hydrate
CDW	construction and demolition waste
COSGB	cemented oil shale residue-steel slag-ground granulated blast furnace slag backfill
CPB	cemented paste backfill
CPV	cumulative pore volume
CSH	calcium silicate hydrate
CT	computed tomography
EDS	energy dispersive spectroscopy
FTIR	fourier transform infrared
GGBS	ground granulated blast furnace slag
LP	limestone powder
LSS	liquid sodium silicate
MIP	mercury intrusion porosimetry
NMR	nuclear magnetic resonance
NS	neutral slag
OPC	ordinary portland cement
OSR	oil shale residue
PG	phosphogypsum
RSM	response surface methodology
SEM	scanning electron microscopy
SH	sodium hydroxide
SS	steel slag
UCS	unconfined compressive strength
WRA	water-reducing admixture
XRD	X-ray diffraction
XRF	X-ray fluorescence
